# Western Eurasian ancestry in modern Siberians based on mitogenomic data

**DOI:** 10.1186/s12862-014-0217-9

**Published:** 2014-10-10

**Authors:** Miroslava Derenko, Boris Malyarchuk, Galina Denisova, Maria Perkova, Andrey Litvinov, Tomasz Grzybowski, Irina Dambueva, Katarzyna Skonieczna, Urszula Rogalla, Iosif Tsybovsky, Ilya Zakharov

**Affiliations:** Institute of Biological Problems of the North, Russian Academy of Sciences, Magadan, Russia; Ludwik Rydygier Collegium Medicum, Institute of Forensic Medicine, Department of Molecular and Forensic Genetics, The Nicolaus Copernicus University, Bydgoszcz, Poland; Institute of General and Experimental Biology, Russian Academy of Sciences, Ulan-Ude, Russia; Scientific and Practical Centre of the State Committee of Forensic Expertises, Minsk, Belarus; Vavilov Institute of General Genetics, Russian Academy of Sciences, Moscow, Russia

**Keywords:** Complete mitochondrial genomes, Western Eurasian mtDNA lineages, Phylogeny, Western Asian ancestry, European ancestry, Northern Asia

## Abstract

**Background:**

Although the genetic heritage of aboriginal Siberians is mostly of eastern Asian ancestry, a substantial western Eurasian component is observed in the majority of northern Asian populations. Traces of at least two migrations into southern Siberia, one from eastern Europe and the other from western Asia/the Caucasus have been detected previously in mitochondrial gene pools of modern Siberians.

**Results:**

We report here 166 new complete mitochondrial DNA (mtDNA) sequences that allow us to expand and re-analyze the available data sets of western Eurasian lineages found in northern Asian populations, define the phylogenetic status of Siberian-specific subclades and search for links between mtDNA haplotypes/subclades and events of human migrations. From a survey of 158 western Eurasian mtDNA genomes found in Siberia we estimate that nearly 40% of them most likely have western Asian and another 29% European ancestry. It is striking that 65 of northern Asian mitogenomes, i.e. ~41%, fall into 19 branches and subclades which can be considered as Siberian-specific being found so far only in Siberian populations. From the coalescence analysis it is evident that the sequence divergence of Siberian-specific subclades was relatively small, corresponding to only 0.6-9.5 kya (using the complete mtDNA rate) and 1–6 kya (coding region rate).

**Conclusions:**

The phylogeographic analysis implies that the western Eurasian founders, giving rise to Siberian specific subclades, may trace their ancestry only to the early and mid-Holocene, though some of genetic lineages may trace their ancestry back to the end of Last Glacial Maximum (LGM). We have not found the modern northern Asians to have western Eurasian genetic components of sufficient antiquity to indicate traces of pre-LGM expansions.

**Electronic supplementary material:**

The online version of this article (doi:10.1186/s12862-014-0217-9) contains supplementary material, which is available to authorized users.

## Background

The peopling of northern Asia by anatomically modern humans probably began more than 40 kya, with the first evidence of human occupation in the Altai region, suggesting the southern mountain belt of Siberia and Middle Siberian plateau was the likely route for this pioneer settlement of northern Asia. Archaeological data definitely supports colonization of the Lake Baikal region in the southern Siberia since the Upper Paleolithic [1–4], however, little is known whether there was biological continuity from around 40 kya to the present. East Asian craniometric features thought to have been derived from early modern East Asians exist in human remains from the Afontova Gora-2 site in upper Yenisei basin and indicate that East Asians moved into southwestern Siberia by 21 kya or even earlier [3]. Yet, the Upper Paleolithic artifacts from the Mal’ta site in the Angara River basin in south-central Siberia (radiocarbon dated to about 23 kya) have been found in association with skeletal remains that bear similar morphology with contemporary anatomically modern humans teeth from Europe thus providing the evidence for links between Siberia and the West during the Upper Paleolithic [1,2,5]. The prevalence of European craniological features among steppe zone inhabitants of Tuva, Altai, and Khakassia of southern Siberia, and even western Mongolia became the most significant since the Bronze Age or even earlier [3,6]. The boundary of the eastern European influence is clearly fixed at Lake Baikal. To the east of Baikal no paleoanthropological findings bear any traces of European admixture [3].

Over the past few years, a number of notable genetic studies on ancient Siberians were conducted. Molecular data suggests that mitochondrial genome of 24 kya old individual from the Mal’ta site belongs to haplogroup U, which has been found at high frequency among Upper Paleolithic and Mesolithic European hunter-gatherers populations, thus testifying a connection between pre-agricultural Europe and Upper Paleolithic Siberia [7]. The western Eurasian mtDNA haplogroup U5a lineages were also revealed in two Neolithic Angara River basin cemetery populations - Kitoi (dated to 7250–6040 years ago) and the Serovo-Glazkovo (dated to 4960–3590 years ago), though the majority of their mtDNAs were of eastern Eurasian ancestry [8]. Notably, the significant influx of western Eurasian mtDNAs into gene pools of ancient Siberians is observed from the early Bronze and Iron Ages (from about 3500 years BC to 200 years AD), thus attesting the eastward migrations of Kurgan people alongside the Eurasian steppe belt, extending from Europe to Manchuria [9–11]. It should be noted however that no genetic data between the Iron Age and today are currently available for Siberian populations.

Evidence from genome-wide autosomal SNPs genotyping testified the presence of both eastern and western Eurasian lineages in gene pools of modern populations of Siberia, a pattern that definitely reflects a complex history of population movements and interactions since Paleolithic times [12–14]. Genome-wide analysis revealed that the genetic landscape of Siberian populations is characterized by two main components unevenly distributed across the studied populations and mixed with other genetic components shared by European or East Asian populations. Admixture dating confirmed that the variable European component seen in the central and northeastern Siberian populations is a result of recent admixture, whereas populations of Altai region of southern Siberia had the highest proportion of European component and the most ancient European admixture dating from the Siberian populations studied [14].

Despite the potential of genomic studies, the particular value of full mitogenome sequencing should be stressed, as the fine genealogical resolution of full mitogenomes together with sufficient sampling can provide a detailed reconstruction of genetic history both for specific lineages and populations in general [15–20]. Thus, for example, the present-day variation of haplogroups C and D, the most frequent throughout northern, eastern, central Asia and America, suggests that these mtDNA clades expanded long before the LGM (dated to 19–26.5 kya), with their oldest lineages being present in the eastern Asia. Unlike in eastern Asia, most of the northern Asian variants began the expansion after the LGM, thus pointing to postglacial re-colonization of northern Asia [17]. Moreover, the results showed that both haplogroups were involved in migrations from eastern Asia and southern Siberia to eastern and northeastern Europe likely during the middle Holocene. In turn, western Eurasian haplogroups found in gene pools of southern Siberians demonstrate an obvious link between populations of Siberia and those of western Asia, the Caucasus, and eastern Europe, with coalescence time estimates suggesting their post-LGM flows from the west [15].

Unfortunately, such issues as timing, origin, and routes of western Eurasian migrations into Siberia remain unresolved mainly due to incompleteness of current complete mtDNA data set. It should be noted that the human mtDNA phylogeny does not equally represent different human populations but it is biased towards representatives of northern and central Europe, so some crucial gaps in certain geographic regions exist. This affects phylogeny of many western Eurasian haplogroups, whose eastern and southern European, Near Eastern and central Asian components are poorly represented. Notwithstanding, recently published comprehensive data set on complete mtDNA variation in Iranians, representative of the majority of the provinces and the ethnic groups [21], as well as global phylogeny reconstruction of western Eurasian haplogroups JT, N1, N2, X, U4, U5, and U8 [22–27] allow us to expand and re-analyze the available data sets of western Eurasian complete mtDNA lineages found in northern Asian populations, reaching 158 mitogenomes (including 85 novel mtDNAs), define the phylogenetic status of Siberian-specific subclades and search for links between mtDNA haplotypes/subclades and events of human migrations.

## Results

It is known that the majority of northern Asian populations exhibit a significant contribution of the western Eurasian mtDNA component, represented by numerous lineages belonging to major haplogroups H, HV, R1, R2, JT, U, N1, N2a, W, and X. Among them, haplogroups H, J, and U are the most frequent. The proportion of western Eurasian lineages is considerably higher in western (21%-70%) and southern (up to 35%) parts of Siberia (reaching their maximum frequencies in populations of Kets, Khanty, Mansi, Altaians and Altaian Kazakhs) than in central (up to 10%) and northeastern (up to 2%) parts of Siberia (Additional file 1 and Additional file 2).

To elucidate the origin of western Eurasian lineages found in mitochondrial gene pools of northern Asians we have analyzed 158 Siberian mitogenomes belonging to different western Eurasian haplogroups, including 85 novel complete mtDNA sequences. The phylogenetic relationships of the 158 western Eurasian mitogenomes found in Siberian populations are depicted in detail in Additional file 3. Information concerning the ethnic origin of each mitogenome, haplogroup affiliation, age estimates, specificity and putative origin of haplogroups and subclades are provided in Additional file 4.

**Haplogroup U** (5.1% overall) is represented in Siberia by almost all major clades, except for U6 and U9 predominant in northern African/Near Eastern populations. **Haplogroups U1 and U7** are very rare in Siberia, but characteristic of western Asian region. Both haplogroups are extremely rare in populations of eastern Siberia (about 0.1%) [15]. In western Siberia, frequency of U1 accounts for 0.2%, but haplogroup U7 is present at relatively high frequency (~7.3%) in Khanty and Mansi gene pools [28,29]. We sequenced one U1 and one U7 complete mtDNA genomes revealed in Buryats and compared them with all published complete sequences, including some Siberian haplotypes available in GenBank. This analysis shows that Siberian lineages belong to subclades U1a1a, U7a2, U7a3 and to a novel subclade U7a4b defined by transition at np 16150, all having a potential western Asian origin (Additional file 5).

Phylogeographic studies have shown that **haplogroup U2e** is present mainly in western Eurasian populations at a frequency of 1% on average, varying from 0.9% in Europe to 1.7% in western Asia [30]. In Siberia, frequency of U2e is about 0.9%, but in some Altaian populations (such as Altaian-Kizhi, Teleuts and Telenghits) this haplogroup reaches higher than the average frequencies (up to 3%) [15,31,32]. However, the Altaian U2e haplotypes are defined by transitions at nps 16214 and 16258 and seem to be unique for Altaians. We have checked this diagnostic motif in all available published data sets on control region mtDNA variation and found similar haplotypes, albeit bearing only 16214 transition, only in populations of northern Caucasus (such as Adygei and Balkars) [33,34].

Until now, the phylogeny of haplogroup U2e has been only partially resolved at the level of complete mtDNAs, especially for eastern European populations. To provide new information concerning the molecular dissection of haplogroup U2e we have completely sequenced 34 samples representing populations of eastern Europe (Russians, Belarusians, Ukrainians, Poles, and Slovaks) and Siberia (Barghuts and Altaian Kazakhs) (Additional file 5). Phylogenetic analysis based on 75 U2e mitogenomes has shown that three clades can be recognized – U2e1, U2e2 and U2e3. We redefined clade U2e2 by the only transition at np 8473 and identified four novel subclades – U2e1a1c, U2e1b1a, U2e1b3 and U2e1i. Analysis demonstrates that Barghut individual together with Russian form subclade U2e1b3, whereas Tubalar and Altaian Kazakh individuals belong to subclade U2e1i, defined by two mutations in control region (at nps 16214 and 16258) and four mutations in the coding region (at nps 2626, 5814, 13914 and 14587). As have already mentioned this subclade appears to be unique, being found only in the Altai region populations. Unfortunately, its molecular dating is impossible because only two identical mitogenomes were sequenced to date. Meanwhile, a coalescence time estimate of the whole clade U2e1 corresponds to ~16.3-19.4 kya, suggesting a relatively early arrival of the U2e1i founder into the Altai region. Note however that the actual appearance of this mtDNA lineage in the Altai could happen much later.

**Haplogroup U3** is typical of the Near East and of the populations from surrounding areas. According to the mtDNA control region data, it is suggested that the U3 was among the main Neolithic founder clades in Europe [33]. However, the reconstruction of complete mtDNA phylogeny and molecular dating of haplogroup U3 subclades is still required, partly due to scarcity of the published complete mtDNA sequences from eastern European populations. Therefore, we provide here new information concerning the 18 mitogenomes of eastern Europeans (Russians, Belarusians, Poles and Czechs), and compare these data with those obtained from western Eurasian populations. Frequency of haplogroup U3 in Siberian populations is very low (0.7%; [15]), so we sequenced the only mitogenome of Altaian individual and found that it together with Hungarian mtDNA belongs to a new subclade U3b2c, for which it is difficult to locate a source now (Additional file 5).

In general, in the phylogenetic tree represented by 81 western Eurasian U3 mitogenomes, we identified two clades (U3a’c and U3b), with many already known in accordance with the PhyloTree data base subclades, except for two new ones - U3b1c and U3b2c. Moreover, phylogeographic analysis along with molecular dating allowed us to recognize two subclades (U3a1 and U3b1b), both presumably originated in Europe relatively recently on the basis of western Asian founders. Subclade U3a1 dated to about 6.4 kya has European-wide distribution, and subclade U3b1b dated to about 2.3 kya seems to be specific for Slavic populations only (Additional file 5).

**Haplogroup U4** is one of the most frequent in populations of eastern Europe, the Volga-Ural region and western Siberia [22,28,29,35–37]. This haplogroup reaches the relatively high frequencies (up to 18%) in aboriginal populations of the Altai-Sayan region – in Altaians and Khakassians [15,19,31,32,38]. To reconstruct phylogeny of Siberian U4-mitogenomes we sequenced 10 mtDNAs of Buryats, Kalmyks, Altaian Kazakhs, Altaians, Shorians, Khakassians and Tuvinians, combined them with 14 mitogenomes retrieved from GenBank (from populations of Yakuts, Tubalars, Mongols, Nganasans, Khants and Mansi) and reconstructed phylogenetic tree based on variation of 125 mitogenomes (Additional file 5). As can be seen, three Siberian lineages fall into eastern European subclades U4a2a and U4d2b, and four - to subclade U4b1b1 having a potentially western Asian source. The remaining northern Asian U4 mtDNAs belong to subclades U4a*, U4a1*, U4a1e, U4a1d, U4b1a4, U4b1*, U4d2*, U4d2a, U4b3a, which origin remains uncertain. Molecular dating of subclades containing Siberian lineages has shown that overwhelming majority of them dates back to the time that does not exceed 6 kya (3.5-3.9 kya for U4a1d, 1.9 kya for U4b1a4, 3.7-5.9 kya for U4b1b1c, 2.6-4.6 kya for U4b3a, 1.5-4.3 kya for U4d2a, 1.5-5.2 kya for U4d2b). Only two haplotypes identified as belonging to the U4a* (in Mansi) and U4b1* (in Tubalars) cannot be dated, but the upper bounds defined by age of the parental clade (11.2-13.9 kya for U4a and 17.2-18.8 kya for U4b1) are within the Upper Paleolithic time scale.

The most ancient European **haplogroup U5** dated to ~25-30 kya is represented in northern Asia by both U5a and U5b clades at frequencies of 0.7% and 0.6%, respectively [15]. To identify U5 haplotypes and clarify their origin we compared 14 complete mitogenomes (including 11 own) from Siberian populations with 225 mtDNAs originating from different Eurasian populations and found that almost all of them belong to already known U5 subclades. The only subclade U5a2a1f harbored previously non-reported diagnostic mutation at np 709 (Additional file 5). Interestingly, Siberian U5b mtDNAs clustered into subclade U5b1b1a, characterized by “Saami-specific” mutation at np 16144, together with Saami, Finns, Russians, Belarusians and Slovaks. Molecular dating results (3.7-4.9 kya) suggest a relatively recent arrival of the U5b1b1a lineage into Siberia.

**Haplogroup U8** is represented by rare clade U8a and U8b, consisting of clades U8b1 and K. U8a is more frequent in Europe, while U8b1 occurs beyond Europe, as it is found in western Asia, the Caucasus and northern Africa. Clade K is more widespread and abundant covering the U8a and U8b1 ranges [26]. In northern Asia, the majority of the U8 mtDNAs belongs to haplogroup K (~1%), whereas U8a and U8b1 accounts for only 0.2% and 0.1%, respectively. In order to identify Siberian U8 lineages we generated 10 K and 1 U8a mitogenomes of aboriginal Siberians and, in addition, 8 U8a and 8 K complete mtDNAs of eastern Europeans (Russians, Poles, Slovaks, Czechs) and compared them with the complete U8 phylogeny, comprising now 971 whole mitogenomes, recently published by Derenko et al. [21] and Costa et al. [26].

**Figure 1:**
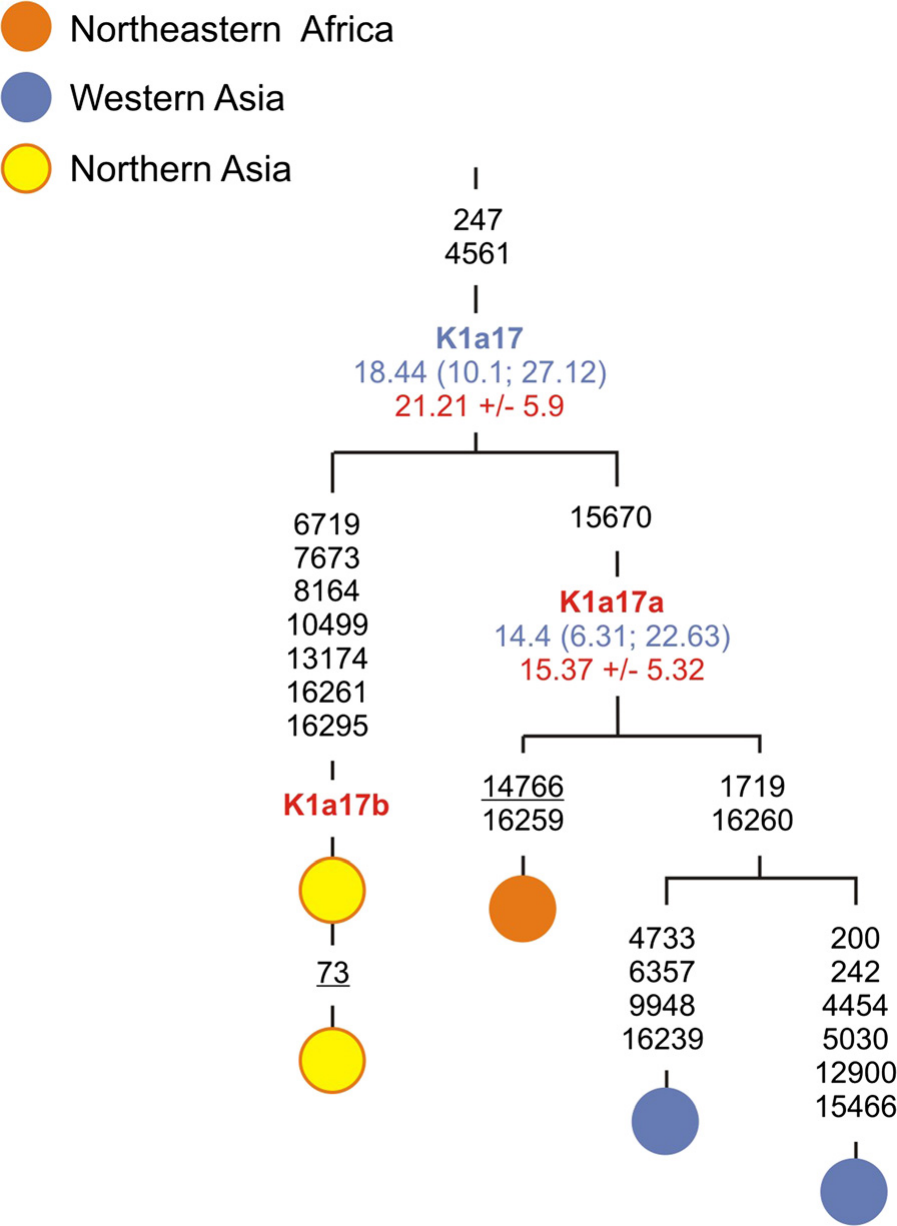
**Phylogenetic tree of haplogroup K1a17.** Numbers along links refer to substitutions scored relative to rCRS [47], back mutation is underlined. Time estimates (in kya) shown along links next to clade labels are based on the complete mtDNA genome clock (given with 95% CI and marked in blue) and the coding region clock (given with the associated standard error and marked in red). Redefined haplogroup label is shown in blue; red are newly identified haplogroups in the present study.

Clade U8a phylogeny demonstrates that our Buryat individual together with eastern Europeans (Russians, Finns and Swedes) belongs to subclade U8a1a1b1. The addition of Polish mtDNA (P14) to the tree gives a new branching point for U8a2, defined now by transitions at nps 827 and 1700. Most surprisingly, by analyzing Siberian K sequences in the context of published Eurasian mtDNAs, we found eight subclades including five new - K1a17b, K1a3b, K1a32, K1b2a2b and K2a5b, two of which (K1a17b and K1a32) seem to be Siberian-specific, being found so far only in Buryats and Khamnigans (Figure 1, Additional file 5).

Using the whole-mitogenome data, we can suppose that Siberian haplogroup K1a17b, K1a3b, and K2a5b lineages have a potential western Asian ancestry, whereas K1b2a2b, K1b2a2*, and K2a3* mitogenomes most likely trace their ancestry within eastern Europe.

In general, out of 58 northern Asian U mitogenomes sequenced to date 25% most likely have eastern European ancestry, and another 22% have a potential western Asian origin.

### Haplogroup H

The control region mtDNA distribution for all northern Asian lineages (n = 5568), summarized in Additional file 1, indicates that the overall frequency of haplogroup H in the northern Asian data base is 3.9%, but this varies from 0.3% in Evens to 14% in Khanty and Mansi. As far as haplogroup H carries only a weak phylogenetic signal in the control region, we cannot emphasize the position of all Siberian haplogroup H lineages in the control region mtDNA data base. We have therefore estimated the fraction of Siberian lineages within haplogroup H that can be allocated to a European or western Asian sources using the 55 Siberian mitogenomes which are currently available (Additional file 4). As can be seen, at least a quarter of Siberian mtDNAs can be assigned to specific subclades within predominantly European haplogroups H1and H3 in the global mitogenome tree (H1a3b, H1b1a, H1b3, H1g1, H1h1, H1*, H1ca, H3g3, and H3*), with another quarter allocated to H2a1a*, H2b, H5a1, H6a1b, H6a1*, H11a1, and H11a2a2. Of these, only H2b, H3g3, and H3* can plausibly be ascribed as having a potential western Asian origin.

There are several minor lineages within haplogroup H (H15a1*, H15a1a1a, H15b, H20a, H35*, and H97), which again nest with western Asian lineages, and further minor lineages belonging to H27a, H7b and H34, which appear to have arisen in Europe or even in eastern Europe. There is, furthermore, an mtDNA from Buryat individual identical to Druze mitogenome, falling alongside with an Italian into haplogroup H7c1, which again most likely derives from a European source. There are also mtDNAs from a single Altaian and Altaian Kazakh, belonging to haplogroup H*, for which it is difficult to locate a source as these branches emerge directly from the root of haplogroup H. H49 also remains ambiguous, though its H49c branch can be considered as Siberian-specific being found in three Yakuts and one Buryat individual only (Additional file 5).

**Figure 2:**
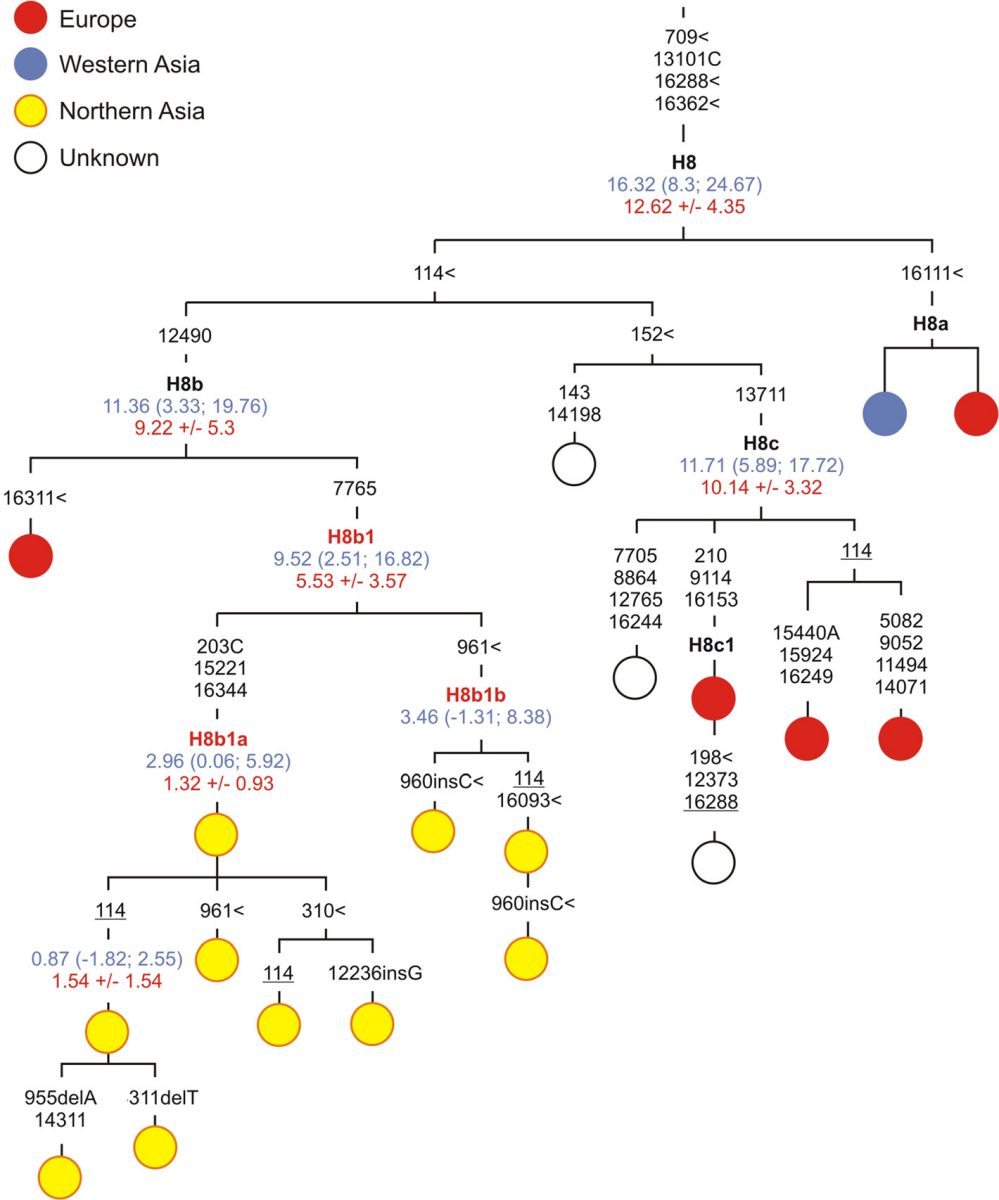
**Phylogenetic tree of haplogroup H8.** Numbers along links refer to substitutions scored relative to rCRS [47]. Transversions are further specified; ins and del denote insertions and deletions of nucleotides, respectively; symbol < denotes parallel mutations, back mutations are underlined. Time estimates (in kya) shown along links next to clade labels are based on the complete mtDNA genome clock (given with 95% CI and marked in blue) and the coding region clock (given with the associated standard error and marked in red). Established haplogroup labels are shown in black; red are newly identified haplogroups in the present study.

Another haplogroup, H8b1 is also found solely in Siberians, and it falls into two distinct sub-clades, H8b1a and H8b1b, with almost the same coalescence age estimated as 0–3.5 kya and 1.3-3 kya, respectively (Figure 2). If we regard H8 as potentially having a Near Eastern and Caucasus source (as has been suggested in [39] based on the mtDNA hypervariable region 1 (HVS 1) variability data), we may assume that H8b1 might have arose *in situ* in southern Siberia after the arrival of the H8b1 founder from somewhere else in the Near East/Caucasus region.

Thus, only 16% of northern Asian haplogroup H lineages are difficult to assign, but at least 42% are likely of European and another 42% of western Asian origin.

### Haplogroups J and T

Haplogroup J comprises 2.1% of the northern Asian control-region data base with frequencies ranging from 0.6% in Yakuts to 12%-15% in Khanty and Mansi. To resolve the haplogroup status and potential origin of northern Asian J-lineages we have analyzed twelve complete mitogenomes which were compared to a global haplogroup J phylogeny reconstructed recently by Pala et al. [25]. There is a sequence shared between Buryat and Italian nesting within western Asian J1b1b1 subclade, and there is also a single Altaian sequence clustering within Iranian-specific subclade J1b1b1a with coalescence age estimated as 6.0-6.6 kya (Additional file 5). Two other Siberian mitogenomes found in Khanty and Mansi belong to J1d6a which again most likely derives from a western Asian source. On the other hand, two Yakut mitogenomes belong to J1c5*, and another to J2a2c1 of likely European origin. The remaining four Siberian J mtDNAs can be assigned to a new J2a2b3 subgroup for which it is difficult to locate a source as these lineages nest together with both northern African-specific J2a2b1 subclade and J2a2b2 lineage of uncertain ancestry originated from the United Kingdom. There is also a single Buryat mitogenome which together with a related sequence from Italy belongs to subclade J1c10a with uncertain origin (Additional file 4 and Additional file 5).

The haplogroup T lineages (1% overall) are more difficult to assign, but at least single Khamnigan mtDNA belonging to T2a1a1b is likely of European origin, whereas the new T2g1a1 subclade specific to Yakuts may have western Asian origin (Additional file 5).

### Other minor western Eurasian mtDNA lineages in northern Asian gene pool

There are numerous other minor western Eurasian mtDNAs in the Siberian mtDNA pool, as can be seen in the control region data base (summarized in Additional file 1), relatively few of which have been subjected to whole-mitogenome sequencing.

**Figure 3:**
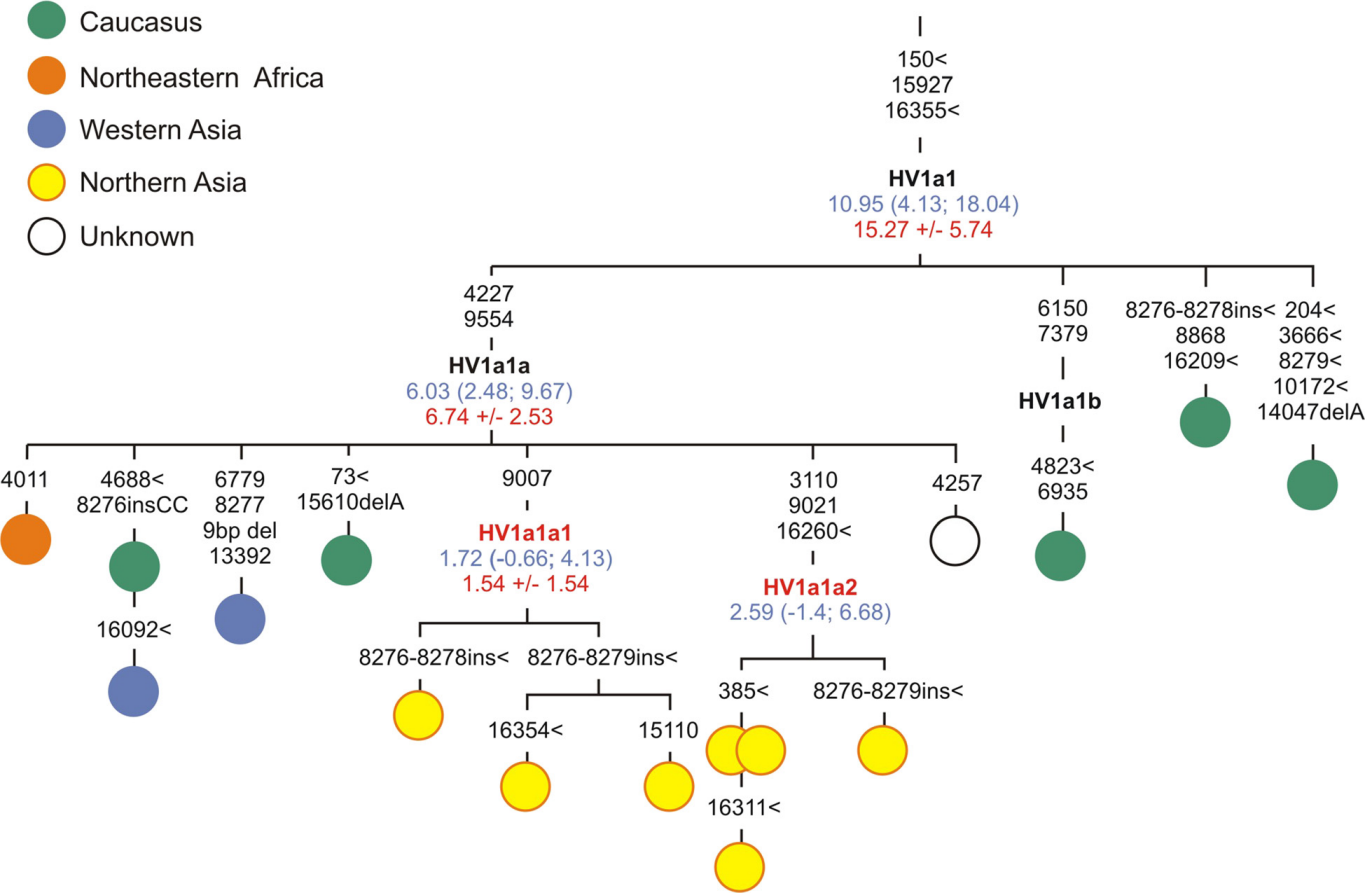
**Phylogenetic tree of haplogroup HV1a1.** Designations are as in Figure 2.

The **haplogroup HV** lineages comprise a number of diverse mtDNAs with overall frequency ~0.6% in the northern Asian control region data base, and eleven of them are completely sequenced to date (Additional file 5). The lineages with a potentially Near Eastern source include HV1, HV12b1 and HV13a. The Siberian-specific HV1a1a1 and HV1a1a2 mitogenomes date to 1.5-2.6 kya and nest within a cluster of Near Eastern/Caucasus HV1a1a lineages dating to 6–6.7 kya (Figure 3, Additional file 5). There are also single haplogroup HV6 and HV9 lineages with a probable European origin.

The **haplogroup W3** lineages all belong to a single subclade W3a1d with a probable European origin. The **haplogroup I** lineages clearly nest within haplogroup I4a – for which an origin cannot be estimated at present.

There are several **haplogroup N1a1a1a1** lineages clustering within Siberian/European subclade N1a1a1a1a with coalescence age estimated as 9.7-10.3 kya (Additional file 5). The control region data base showed that the N1a1a1a1a clade, characterized by 16189 in HVS1, is found in Kazakhstan, Altai and Baikal regions of southern Siberia and eastern Europe, so its origin remains uncertain [40]. There is single Buryat mtDNA belonging to redefined haplogroup N1a1b1a1 of likely western Asian origin.

The **haplogroup X** mtDNAs found in Altai and Baikal regions of southern Siberia all belong to X2e2a1 subclade nesting within X2e2a which includes lineages from Near East and Caucasus, pointing thus to western Asian ancestry of Siberian X2e2a1 lineages (Additional file 5).

Overall, from a survey of 158 western Eurasian mtDNA genomes found in Siberia we estimate that 37% of them most likely have western Asian and another 29% European ancestry, whereas 34% remain ambiguous. It is striking that 65 of northern Asian mitogenomes, i.e. ~41%, fall into 19 branches and subclades which can be considered as Siberian-specific being found so far only in Siberian populations. From the coalescence analysis it is evident that the sequence divergence of Siberian-specific subclades was relatively small, corresponding to only 0.6-9.5 kya (using the complete mtDNA rate) and 1–6 kya (coding region rate), thus implying a Holocene origin and expansion of these lineages in northern Asia (Table 1).

**Table 1:** **Age estimates for Siberian-specific western Eurasian mtDNA subclades**

**Clade**	**No. of mtDNAs**	**Age estimates in kya**	
		**Complete genome rate (95** **%** **CI)** ^**a**^	**Coding region rate ± s.e.** ^**b**^
U4b1a4	3	1.93 (−0.25; 4.16)	0
U4b1b1	4	3.71 (0.79; 6.68)	5.93 ± 2.55
U4b3a	3	2.59 (−0.34; 5.57)	4.61 ± 2.66
U4d2a	3	4.33 (0.53; 8.24)	1.54 ± 1.54
H8b1	10	9.52 (2.51; 16.82)	5.53 ± 3.57
> H8b1a	7	2.96 (0.06; 5.92)	1.32 ± 0.93
> H8b1b	3	3.46 (−1.31; 8.38)	0
HV1a1a1	3	1.72 (−0.66; 4.13)	1.54 ± 1.54
HV1a1a2	4	2.59 (−1.4; 6.68)	0
J2a2b3	4	0.64 (−0.61; 1.91)	1.15 ± 1.15
T2g1a1a	3	0.86 (−0.82; 2.55)	0
X2e2a1	5	1.55 (−0.71; 3.83)	2.77 ± 2.06

^a^Mutation rate is one mutation per every 3624 years [54].

^b^Mutation rate is one mutation per every 4610 years [55].

## Discussion

In the last few years, the availability of a growing number of complete mitogenomes (20,666 in PhyloTree data base; released on February 19, 2014) has considerably improved our knowledge on the worldwide human mtDNA phylogeny [41]. The numerous novel subclades were characterized by more distinct geographical distributions, thus allowing inferences on demographic events that occurred at both regional and continental levels [21–27]. In this study, we aimed to define the exact phylogenetic status of western Eurasian mitogenomes specific for northern Asian populations, which are rather uncommon and not well-represented in the current complete mtDNA data set, and pinpoint the source for those lineages by applying the phylogeographic approach. It has been shown that this kind of analysis can be very powerful, because nesting of particular mitogenomes within clusters from a specific geographical region makes it possible to reveal the origin of those lineages, by applying the parsimony principle [26,42].

Although the genetic heritage of aboriginal Siberians is mostly of eastern Asian ancestry, analyses of autosomal SNP data and uniparental markers show that a substantial western Eurasian component is observed in the majority of northern Asian populations. Thus, the composition of mtDNA lineages of western Eurasian origin revealed in southern Siberian populations suggests that there were at least two migrations into southern Siberia, one from eastern Europe and the other from western Asia/the Caucasus. Traces of both migrations associated with different mtDNA haplogroups were detected in all southern Siberian regional groups, with minor influence on the most northeastern of the eastern Sayan populations [15]. However, the extent to which modern Siberians trace their western Eurasian ancestry to the eastern Europe or to western Asia/Caucasus remains unclear.

Our results, primarily from the detailed analysis of 158 Siberian-specific western Eurasian mtDNA genomes, suggest that nearly 40% of western Eurasian maternal lineages found in northern Asia trace their ancestry to western Asia, whereas another 30% most likely have European ancestry. Our analysis shows that at least one of the major Siberian haplogroup H mtDNA lineages, H8b1, has a deep western Asian ancestry, tracing back at least as far as the early or mid-Holocene. Furthermore, our results suggest that two newly described southern Siberian haplogroup HV subclades, HV1a1a1 and HV1a1a2, for which western Asian ancestry is also proposed, dated back to late and mid-Holocene, respectively. Despite some uncertainty in its ancestral branching relationship, a western Asian ancestry seems likely for the U4b1b1c subclade found so far in southern (Altaians, Shorians) and western (Mansi) Siberia as well as in Iran and the Volga-Ural region. This subclade dates to ~4-6 kya, thus suggesting a mid-Holocene radiation in Siberia. Another Siberian specific subclade, X2e2a1, dates to ~1.5-3 kya and nests within deeper Near Eastern lineages dating to ~5 kya, also suggesting (similarly to U4b1b1c) the mid-Holocene dispersal from Near East to southern Siberia. Aside from this, there is a single highly divergent K1a17b-lineage from Baikal region populations nested within western Asian subclade K1a17, pointing to a gene flow from western Asia to southern Siberia, which might have occurred at the end of LGM but not earlier than 18–21 kya. The phylogenetic nesting patterns suggest that several minor lineages may have been introduced in Siberia at the same time (with some lost later by drift). Thus for example, U7a2*, HV13a, and N1a1b1a1 mtDNAs, with their nesting within preliminary western Asian lineages, were most likely assimilated not earlier than 18.3-24.2 kya, whereas U2e1i southern Siberian founder having a putative Caucasus origin may have been introduced into northern Asia later, ~ 15.8-16.6 kya. In turn, U4a1e, U4a1d, H6a1, HV6, and HV9 mtDNAs with their nesting within preliminary European lineages may have been introduced into northern Asia even later, in the time range from ~ 9.3 to ~14.2 kya.

## Conclusions

Overall, the phylogeographic analysis strongly implies that the western Eurasian founders, giving rise to Siberian specific subclades, trace their ancestry only to the early and mid-Holocene, though some of genetic lineages may trace their ancestry back to the end of LGM. Importantly, we have not found the modern northern Asians to have western Eurasian genetic components of sufficient antiquity to indicate traces of pre-LGM expansions, that originated from the Upper Paleolithic industries present both in the southern Siberia and Siberian Arctic, and that date back to ~30 kya, well before the LGM [43–45]. Apparently, the Upper Paleolithic population of northern Asia, whose western Eurasian ancestry was approved recently by complete genome sequencing of 24 kya-old individual from Mal’ta and 17 kya-old individual from Afontova Gora in south-central Siberia, did not leave a genetic mark on the female lineages of modern Siberians. It is probable that the initial population expansion in the southern Siberia region involved maternal lineages other than present now, or that there was a substantial gene flow into the region after the LGM, most probably from eastern Asian sources as have been suggested by Raghavan et al. [7].

Further complete mitogenome and complete genome-based studies of ancient northern Asian specimens will be extremely informative for revealing spatial patterns attributable not only to primary colonization events but also to more-recent migrations.

## Methods

### Ethics statement

The study was approved by the Ethics Committee of the Institute of Biological Problems of the North, Russian Academy of Sciences, Magadan, Russia (statement no. 003/012 from 15 March, 2012). All subjects provided written informed consent for the collection of samples and subsequent analysis.

### Mitochondrial genome sequencing

Out of about 5700 samples of northern, eastern and western Asians as well as Europeans that were screened previously for haplogroup-diagnostic RFLP markers and subjected to control region sequencing (Additional file 6) a total of 166 samples were selected for complete mtDNA sequencing (Additional file 7). Samples were selected to include the widest possible range of western Eurasian mtDNA lineages found in Siberian populations as well as some European mtDNAs belonging to sub-haplogroups U2, U3, U4, U8a, K, and H, which are still underrepresented in the published data sets on complete mtDNA variation. The complete mtDNA sequencing was performed as described in detail by Torroni et al. [46] using an ABI 3130 and ABI 3500xL Genetic Analyzers. DNA sequence data were analyzed using SeqScape 2.5 software (Applied Biosystems) and compared to the revised Cambridge reference sequence (rCRS) [47]. The RSRS [48] was not used in the analysis due to potential errors which it contains as shown by Malyarchuk [49]. Besides, switching to a new reference sequence (from rCRS to RSRS) in the mtDNA studies still has not found an international consensus [50,51].

### Statistical analysis and molecular dating

For reconstruction of the mtDNA phylogenies the data obtained in this study and those published recently [20,21,26] as well as all available at PhyloTree data base [41] were taken into account. A nomenclature, which we hereby update follows [41], with several new modifications. The most-parsimonious trees of the complete mtDNA sequences were reconstructed manually, and verified by means of the Network 4.5.1.0 software [52], and using mtPhyl software (http://eltsov.org), which is designed to reconstruct maximum parsimony phylogenetic trees. Both applications calculate haplogroup divergence estimates (rho) and their error ranges, as average number of substitutions in mtDNA clusters (haplogroups) from the ancestral sequence type [53]. Values of mutation rates based on mtDNA complete genome variability data (one mutation every 3624 years [54] and coding region substitutions (one mutation every 4610 years [55]) were used. Nucleotide position (np) 16519 as well as positions showing point indels and/or transversions located between nps 16180–16193, 303–315, 522–524, 573–576 were excluded from the phylogenetic analysis.

## Availability of supporting data

The data set supporting the results of this article is included within the article and its additional files. The 166 novel complete mtDNA sequences supporting the results of this article are available in the National Center for Biotechnology Information (Genbank) under accession numbers KJ856675-KJ856840, http://www.ncbi.nlm.nih.gov/Genbank/. The assignment of all samples to their sequence accession numbers is listed in Additional file 7.

## Additional files

Additional file 1:
**Western Eurasian mtDNA haplogroup** **frequencies in northern Asian populations (%).**
Additional file 2:
**Map showing the locations of the northern Asian populations divided into seven regional groups color coded as: northeastern Siberia – blue, western Siberia – red, central Siberia – green, Altai region of southern Siberia – orange, eastern Sayan region of southern Siberia – green mint, Baikal region of southern Siberia – yellow, Okhotsk/Amur region of southern Siberia – purple.** The total frequency (%) of western Eurasian mtDNA lineages in each population is given in circles. Additional file 3:
**Maximum-parsimony phylogenetic tree of 158 complete mtDNA sequences, constructed using the program mtPhyl.** Numbers along links refer to substitutions scored relative to the rCRS (Andrews et al. [47]). Transversions are further specified; ins and del denote insertions and deletions of nucleotides, respectively; back mutations are underlined; symbol < denotes parallel mutations; heteroplasmies are labeled using the IUPAC code. For phylogeny reconstruction, the length variation in the poly-C stretches at nps 303–315 and 16184–16194 was not used. Polymorphism at np 16519 and A-C transversions at nps 16182 and 16183 were excluded. Samples labeled as in Additional file 4 and Additional file 7 and newly sequenced samples are marked in red. Established haplogroup labels are shown in black; blue are redefined and red are newly identified haplogroups in the present study. Additional file 4:
**Population origin, haplogroup affiliation, specificity and putative origin of western Eurasian northern Asian mitogenomes.**
Additional file 5:
**Maximum-parsimony phylogenetic trees of complete mtDNA sequences belonging to haplogroups U1a, U7, U2e, U3, U4, U5a2a, U5b1b1a, U8a, K1a, K1a17, K1b2, K2a3, K2a5, H11, H15, H20, H8, H49, H7c, H97, JT, HV, N1a1, W3, and X2e2, constructed using the program mtPhyl.** Numbers along links refer to substitutions scored relative to rCRS (Andrews et al. [47]). Transversions are further specified; ins and del denote insertions and deletions of nucleotides, respectively; back mutations are underlined; symbol < denotes parallel mutation; heteroplasmies are labeled using the IUPAC code. Newly sequenced Siberian (marked in yellow) and European (marked in green) samples labeled as in Additional file 4 and Additional file 7, for published data the accession number in indicated. Coalescence time estimates expressed in kya are shown along links next to clade labels and were calculated by computing the averaged distance (rho) and the standard error (s) (Saillard et al. [53]). Calculations were obtained using the entire mtDNA genomes but excluding the length variation in the poly-C stretches at nps 303–315 and 16184–16194 and hot spot mutations such as 16182C, 16183C, and 16519. Values of mutation rates based on mtDNA complete genome variability data (Soares et al. [54]; marked in blue) and coding region substitutions (Perego et al. [55]; marked in red) were used. Established haplogroup labels are shown in black; blue are redefined and red are newly identified haplogroups in the present study. Additional file 6:
**List of population samples subjected previously for** **haplogroup-diagnostic** **RFLP screening and control region sequencing from where 166 samples were selected for complete mtDNA sequencing.**
Additional file 7:
**Origin and** **sub-haplogroup** **affiliation of complete** **mtDNAs sequenced in this study.**
