# Supplementary figures and images for: Western Eurasian ancestry in modern Siberians based on mitogenomic data
Source: BMC Evol Biol. 2014 Oct 10;14:217. doi: 10.1186/s12862-014-0217-9 (PMC4195960; doi:10.1186/s12862-014-0217-9)

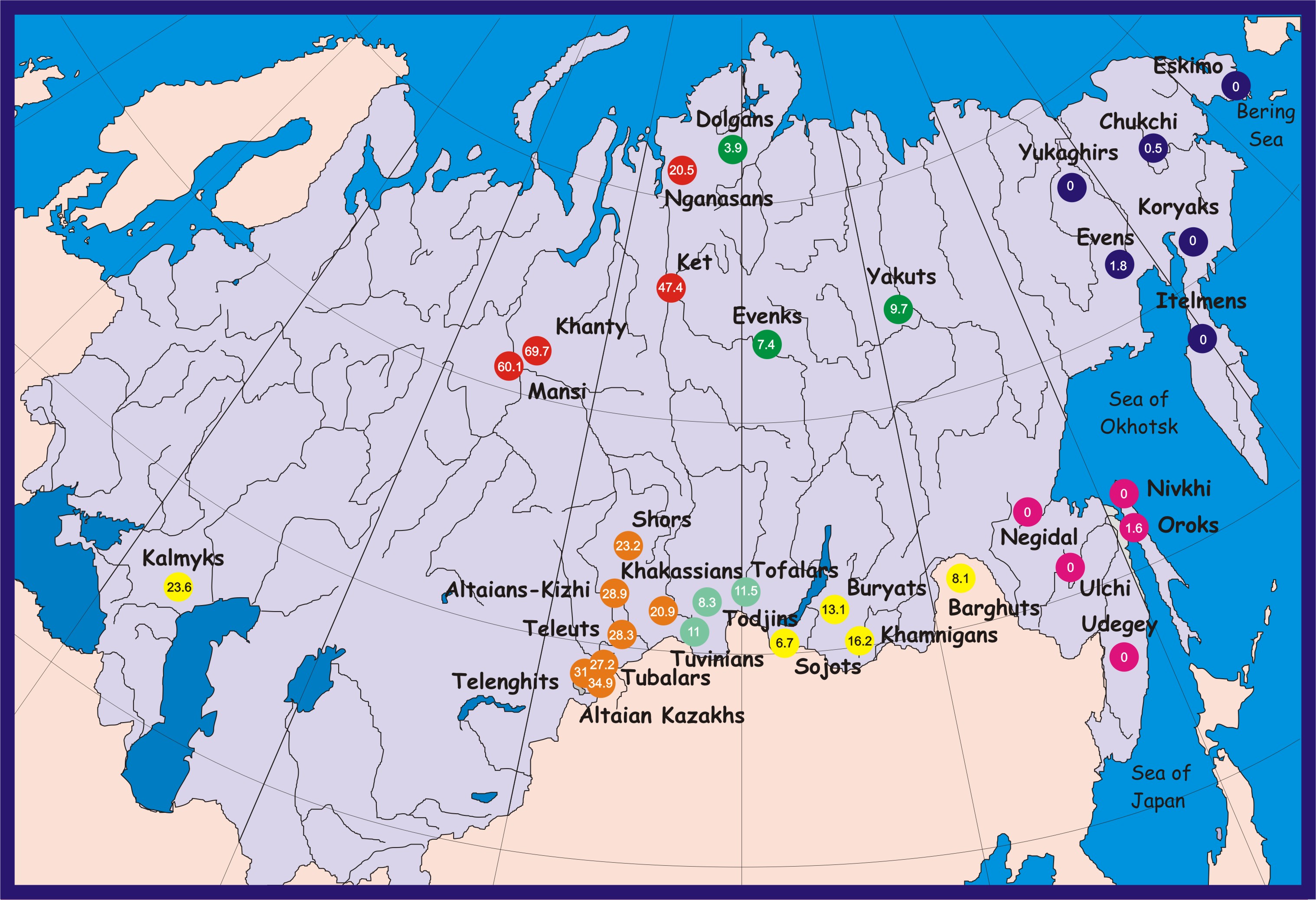

Supplement: Additional file 2: — Map showing the locations of the northern Asian populations divided into seven regional groups color coded as: northeastern Siberia – blue, western Siberia – red, central Siberia – green, Altai region of southern Siberia – orange, eastern Sayan region of southern Siberia – green mint, Baikal region of southern Siberia – yellow, Okhotsk/Amur region of southern Siberia – purple. The total frequency (%) of western Eurasian mtDNA lineages in each population is given in circles. [file 12862_2014_217_MOESM2_ESM.jpeg]
